# Acute high-intensity exercise alters gut microbiota composition and energy metabolism in different strains of mice

**DOI:** 10.3389/fmicb.2026.1790697

**Published:** 2026-04-22

**Authors:** Ruolin Gao, Jie Wang, Jiajia Song, Wenlong Zhang, Lei Quan

**Affiliations:** Tianjin Key Laboratory of Exercise Physiology and Sports Medicine, School of Exercise and Health Sciences, Tianjin University of Sport, Tianjin, China

**Keywords:** acute high-intensity exercise, energy metabolism, gut microbiota, *Muribaculum*, temporal dynamics

## Abstract

**Introduction:**

The gut microbiota is a critical modulator of host energy metabolism and immune regulation. Although exercise modulates microbial composition, the temporal dynamics and strain-specific impact of acute high-intensity exercise remains unclear.

**Methods:**

This study investigated dynamic alterations in gut microbiota following acute high-intensity exercise in BALB/c and C57BL/6 mice, focusing on energy and gut health-related genera. Age-matched male mice underwent a 30-min high-intensity treadmill run. To capture temporal dynamics, colonic content samples were collected at 0, 30, and 60 min post-exercise for 16S rRNA gene sequencing.

**Results:**

Exercise induced significant remodeling of gut microbiota composition in both strains, with a notable post-exercise elevation in the *Bacteroidetes/Firmicutes* ratio, particularly in C57BL/6 mice (C57Cr vs. C57T0, *p* < 0.01; C57Cr vs. C57T60, *p* < 0.05; BCCr vs. BCT30, *p* < 0.05). Both alpha and beta diversity metrics revealed significant exercise-induced microbial changes, with C57BL/6 mice showing a more pronounced increase in diversity at 30 and 60 min post-exercise (OTUs and observed species, all *p* < 0.001) compared to BALB/c mice. Functional analysis revealed strain-specific responses: BALB/c mice exhibited upregulated enzymes involved in energy metabolism but impaired immune regulation, indicating compromised intestinal barrier integrity and delayed energy recovery. In contrast, C57BL/6 mice displayed enhanced anti-inflammatory capacity, accelerated energy recovery through enrichment of energy metabolism-related genera, and maintained gut integrity through the proliferation of beneficial bacteria. The potential important genus *Muribaculum* identified as a key exercise-responsive taxon across time points, potentially associated with gut motility. The role of *Muribaculum* warrants further using multi-omics investigation.

**Discussion:**

These findings provide a foundation for identifying genetic loci influencing exercise microbiota interactions, thereby providing a theoretical basis for microbiota-based personalized exercise interventions.

## 1 Introduction

The gut microbiota comprises a complex ecosystem of trillions of microorganisms that critically influence host physiology and susceptibility to diseases. Recognized as a novel endocrine organ (Jiang, 2024), the gut microbiome possesses collective genetic potential termed the gut metagenome (Sales and Reimer, 2023). Compared to the human genome, which encodes approximately 23,000 genes, the gut microbiome contains approximately 3.3 million genes involved in modulating numerous aspects of host function, including energy extraction and metabolism (Turnbaugh et al., 2006), inflammation (Boulangé et al., 2016), vitamin synthesis (Wan et al., 2022), and immunity (Mohr et al., 2020). The inherent plasticity of the gut microbiota further positions it as a promising therapeutic target for disease prevention and management (Gomez et al., 2019). As a pivotal interface between the host and external environment, the composition and functional output of the gut microbiota are influenced by various extrinsic factors, including diet and exercise. Disruption of the homeostatic host-microbiota interaction has been established as a driver of multiple diseases (Cani and Van Hul, 2024). Exercise has emerged as a powerful modulator of both the composition and metabolic activity of the gut microbiota (Monda et al., 2017; Hao et al., 2022; Cho et al., 2020; Lkhagva et al., 2021; Pereira et al., 2025; Morgado et al., 2023). Research aimed at understanding the interaction between exercise and gut microbiota has largely expanded within the past decade. In 2014, Clarke and colleagues published a landmark study demonstrating that professional rugby players exhibited higher alpha diversity and a greater relative abundance of the health-associated genus *Akkermansia*, compared to sedentary controls with high or low body mass indices (Clarke et al., 2014). These findings stimulated numerous subsequent investigations into the exercise-gut microbiota relationship. Several observational studies have subsequently reported that exercise-associated states correlate with increased alpha diversity (Estaki et al., 2016; Mörkl et al., 2017), enrichment of beneficial microbial taxa (Estaki et al., 2016; Jang et al., 2019; O'Donovan et al., 2020), and elevated fecal concentrations of short-chain fatty acids (SCFAs) (Barton et al., 2018). This bidirectional relationship is supported by findings indicating that the gut microbiota and its metabolites can reciprocally affect exercise capacity and performance by enhancing lactate metabolism, increasing glycogen storage, and altering substrate metabolism in skeletal muscle (Sales and Reimer, 2023). Specific microbial-derived SCFAs, such as acetate and propionate, have also been directly implicated in improved athletic performance (Scheiman et al., 2019; Koh et al., 2016). However, whether and how gut microbiota alterations directly mediate the metabolic benefits associated with exercise remain unclear.

The effect of exercise on gut microbiota also depends on exercise modality and intensity. Aerobic exercise has been shown to induce diet-independent compositional and functional changes in the human gut microbiota (Allen et al., 2018b), which reverse upon cessation of training (Allen et al., 2018b; Hampton-Marcell et al., 2020). For example, high-intensity intermittent exercise has been reported to increase the abundance of *Bacteroides*, the *Bacteroidetes*-to-*Firmicutes* ratio, and alpha diversity, suggesting a potential to counter dysbiosis associated with diet-induced obesity and various metabolic disorders (Denou et al., 2016). Chronic exercise beneficially modulates the gut microbiota and its metabolic output. The positive effects of chronic exercise on gut microbiota may depend on acute changes in microbial community structure and function following a single exercise session (acute exercise). Thus, delineating the dynamic changes in gut microbiota following acute exercise serves as a critical prerequisite for unraveling the microbial mechanisms underlying the health benefits of exercise, ultimately, for optimizing evidence-based exercise interventions.

A significant challenge in translating findings from murine models to humans stems from the greater inter-subject variability in human gut microbiome composition (Tanes et al., 2021). For instance, dietary interventions typically produce more substantial alterations in gut microbiota composition in mice compared to humans (Baxter et al., 2019; Johnson et al., 2019; Wu et al., 2011). To identify robust and conserved exercise-responsive microbial signatures, we integrated time-series datasets from BALB/c and C57BL/6J mice, two widely utilized laboratory mouse strains. By examining the differential responses of gut microbiota to acute high-intensity exercise across distinct genetic backgrounds, we aimed to delineate potential important exercise-responsive taxa and achieve high-resolution temporal insights into gut microbiota dynamics post-exercise.

In this study, we subjected C57BL/6 and BALB/c mice to a single bout of acute high-intensity exercise, in order to examine strain-specific alterations in gut microbiota and differences between strains following exercise intervention. While previous research relying on single-time-point sampling has demonstrated exercise-induced structural changes in gut microbiota, such approaches are limited in capturing transient microbial dynamics. We hypothesize that post-exercise sampling time points are critical for capturing acute exercise-induced microbial shifts. Thus, to investigate temporal alterations in gut microbiota across two mouse strains with different motility characteristics following acute high-intensity exercise, samples were collected at multiple time points (0, 30, and 60 min) at rest and following a single acute high-intensity exercise session.

## 2 Materials and methods

### 2.1 Animal and experimental design

Eight-week-old male BALB/c mice (*n* = 32) and C57BL/6J mice (*n* = 32), weighing 27 ± 2 g, were purchased from Charles River Co. (Beijing, China). All mice were housed individually in cages in a controlled environment (23 ± 1 °C, 12:12 h light–dark cycle). All shipped mice were acclimated to the new controlled surroundings for 1 week. To minimize the variation of environmental factors, all mice had ad libitum access to autoclaved water and were fed a standard commercial rodent chow diet (Rodent breeding diet, code 1035, Beijing HFK Bioscience Co., Ltd., Beijing, China).

All exercise experiments were conducted at the same time of the day (between 9 a.m. and 12 a.m., 1–3 h after lights off), with immediate sample processing to control for circadian variation. Exercise protocol was adapted from standardized running protocols used in previous studies with slight modifications. The protocol was designed in line with PASS protocols of the Molecular Transducers of Physical Activity Consortium (MoTrPAC) (MoTrPAC Study Group, Lead Analysts, MoTrPAC Study Group, 2024; Sanford et al., 2020; Sato et al., 2022). Sampling series was selected towards early time points (0, 30, and 60 min post-exercise) to capture the temporal dynamics of acute-exercise responses.

### 2.2 Exercise protocol

All experiments were conducted between 9 a.m. and 12 p.m. in non-fasted animals. A modified exercise protocol based on previous studies was employed (Sanford et al., 2020; Sato et al., 2022). In brief, mice were habituated to treadmill running (Model DB030, Beijing Zhishuduobao Biological Technology, Beijing, China) following a 5-day acclimatization protocol as described below (Days 1–5):

Day 1 (15 min total; 5 deg. inclination).

For the first 5 min, mice were placed on the stationary treadmill.

Start running at 6 m/min and accelerate by 2 m/min every 2 min up to 12 m/min.

Day 2 (15 min total; 5 deg. inclination).

Start running at 6 m/min and accelerate by 2 m/min every 2 min up to 14 m/min.

Day 3 (15 min total; 5 deg. inclination).

Start running at 6 m/min and accelerate by 2 m/min every 2 min up to 16 m/min.

Day 4 and Day 5.

Rest.

Mice completed the acclimatization protocol were randomly divided into two groups in each mouse strain. On the day of study, food was removed from all the cages at 8 a.m. (1 h prior to the experiment). The exercise group of mice were subjected to a single bout of acute treadmill running as described below (Day 6), whereas the control group of mice were placed on a stationary treadmill alongside the running treadmill for the same amount of time (sham-exercise). To ensure exercise-specific effects, control mice were subjected to identical handling, environmental, and procedural conditions as exercised mice, except for treadmill belt movement.

Day 6 (30 min total; 5 deg. inclination) Start running at 6 m/min and accelerate by 2 m/min every 2 min up to 20 m/min, an intensity previously estimated to correspond to approximately 80–90% VO_2_max in mice based on published regression equations (Qin et al., 2024; Sousa et al., 2019; Xu L. et al., 2023). Mice attempting to rest were encouraged to continue running by gently tapping on their back.

**Figure 1:**
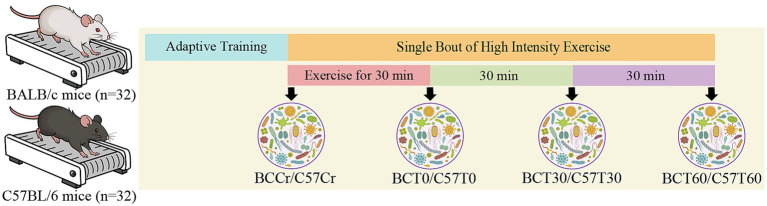
Study design.

Mice were kept in their host cages with no access to food between the end of exercise and before they were sacrificed by cervical dislocation immediately, 30 or 60 min after the exercise bout (n = 8 for each time point per strain) or immediately after shamexercise (*n* = 8), respectively (Figure 1).

### 2.3 Sample acquisition

#### 2.3.1 Fecal sample collection

In both BALB/c mice and C57BL/6 J mice, samples were aseptically collected at immediately, 30 or 60 min after the exercise bout or immediately after sham-exercise as baseline. The entire intestine was removed and colonic segments were excised using a sterile scalpel at 4 °C. To ensure consistency in microbiome analysis, the colonic contents were gently extruded in their entirety from the whole colon using sterile tweezers, avoiding cross-contamination, and placed into sterile centrifuge tubes. In order to protect the quality of the samples, the samples were kept at −80 °C.

#### 2.3.2 Extraction of fecal sample DNA and quality control

The CTAB/SDS method was used to extract total genome DNA from fecal samples, using the CTAB extraction buffer (N0211, NobleRyder, Beijing, China). All samples from the same strain were processed in parallel to minimize batch effects. The purity and concentration of the DNA was detected by agarose gel electrophoresis, and the appropriate amount of DNA was placed in a centrifuge tube and diluted to 1 ng/μL with sterile water. Both extraction blanks (sterile water subjected to the full DNA extraction protocol) and PCR-negative controls (nuclease-free water replacing template DNA) were included to assess contamination during DNA extraction and amplification steps.

### 2.4 16S rRNA gene sequencing and data processing

#### 2.4.1 16S rRNA gene amplification and library preparation

The diluted genomic DNA was used as a template for PCR amplification using specific primers, high efficiency high fidelity enzyme and Phusion® High-Fidelity PCR Master Mix with GC Buffer to ensure the amplification efficiency and accuracy. Primers in the 16S V4 region (515F and 806R) identified bacterial diversity; primers in the 18S V4 region (528F and 706R) identified prokaryotic microbial diversity; and primers in the ITS1 region (ITS5-1737F and ITS2-2043R) identified fungal diversity. In addition, amplified regions included: 16S V3-V4/16S V4-V5/16SV5-V7; Archaea 16S V4-V5/Archaea 16S V8; 18S V9 and ITS2 regions. For electrophoresis detection, screen out eligible PCR products using an agarose gel at a 2% concentration. After being purified using magnetic beads, the qualified PCR products were quantified using enzyme labeling and mixed in aliquots based on their concentration. Following mixing, they were electrophoretically detected using a 2% agarose gel once more, and the target bands were recovered using the Qiagen gel recovery kit. The TruSeq® DNA PCR-Free Sample Preparation Kit was used to create the library, Qubit and Q-PCR were used to quantify it, and NovaSeq6000 was used to qualify it for online sequencing.

#### 2.4.2 Paired-end reads assembly and quality control

Paired-end reads were assigned to samples based on their unique barcode and truncated by cutting off the barcode and primer sequence. Paired-end reads were merged using FLASH (VI.2.7^1^) (Magoč and Salzberg, 2011), which was designed to merge paired-end reads when at least some of the reads overlap the read generated from the opposite end of the same DNA fragment, and the splicing sequences were called raw tags. Quality filtering on the raw tags were performed under specific filtering conditions to obtain the high-quality clean tag (Bokulich et al., 2013) according to the QIIME (V1.9.1^2^) (Caporaso et al., 2010) quality controlled process. The tags were compared with the reference database (Silva database^3^) using UCHIME algorithm (UCHIME Algorithm^4^) (Edgar et al., 2011) to detect chimera sequences, and then the chimera sequences were removed (Haas et al., 2011). Then the Effective Tags finally obtained.

#### 2.4.3 Bioinformatics analysis

Based on the valid data, species categorization analysis and OTU (Operational Taxonomic Unit) clustering were then carried out. The OTUs sequences were classified by species annotation according to the silva SSUrRNA database (Quast et al., 2013). To assess the diversity of species complexity in the samples, Alpha diversity analysis was performed by observed species index and Shannon index, and Beta diversity was analyzed by Principal Coordinates Analysis (PCoA) performed based on UniFrac distances. To statistically test for differences in microbial community structure between groups, we performed analysis of similarities (ANOSIM) and permutational multivariate analysis of variance (PERMANOVA) using the vegan package in R (version 4.4.2) based on Bray-Curtis distances, each with 999 permutations. Differences in abundance between groups were calculated using the R (version 4.4.2) edgeR package, and heatmap and Venn plots were plotted. All difference abundance histograms were plotted by LDA Effect Size (LEfSe) analysis performed using the R (version 4.4.2) lefse package implementation, with a linear divergence analysis (LDA) score threshold set at 4.0. LDA scores indicate the degree of effect of significantly different species between groups, with higher scores indicating higher scores indicate greater differences in characteristics between the two groups. Functional predictions of the differentially abundant genera were derived from previously published literature.

### 2.5 Statistical analysis

The analyses were performed using R (version 4.4.2) and SPSS (version 26.0) and expressed as means ± SEM. After data were tested for normality, the Student’s t test was used to compare two groups. For comparisons involving more than two groups, one-way analysis of variance (ANOVA) was performed, followed by post-hoc pairwise comparisons using the least significant difference (LSD) test. To control the false discovery rate due to multiple testing, *p*-values from all post-hoc comparisons were adjusted using the Benjamini–Hochberg procedure (FDR < 0.05). Data differences were considered to be statistically significant at a value of *p* < 0.05. Figures were generated using the GraphPad Prism software (Version 9.0; San Diego, CA, USA).

## 3 Results

### 3.1 Overall distribution of gut microbiota species in response to acute exercise

**Figure 2:**
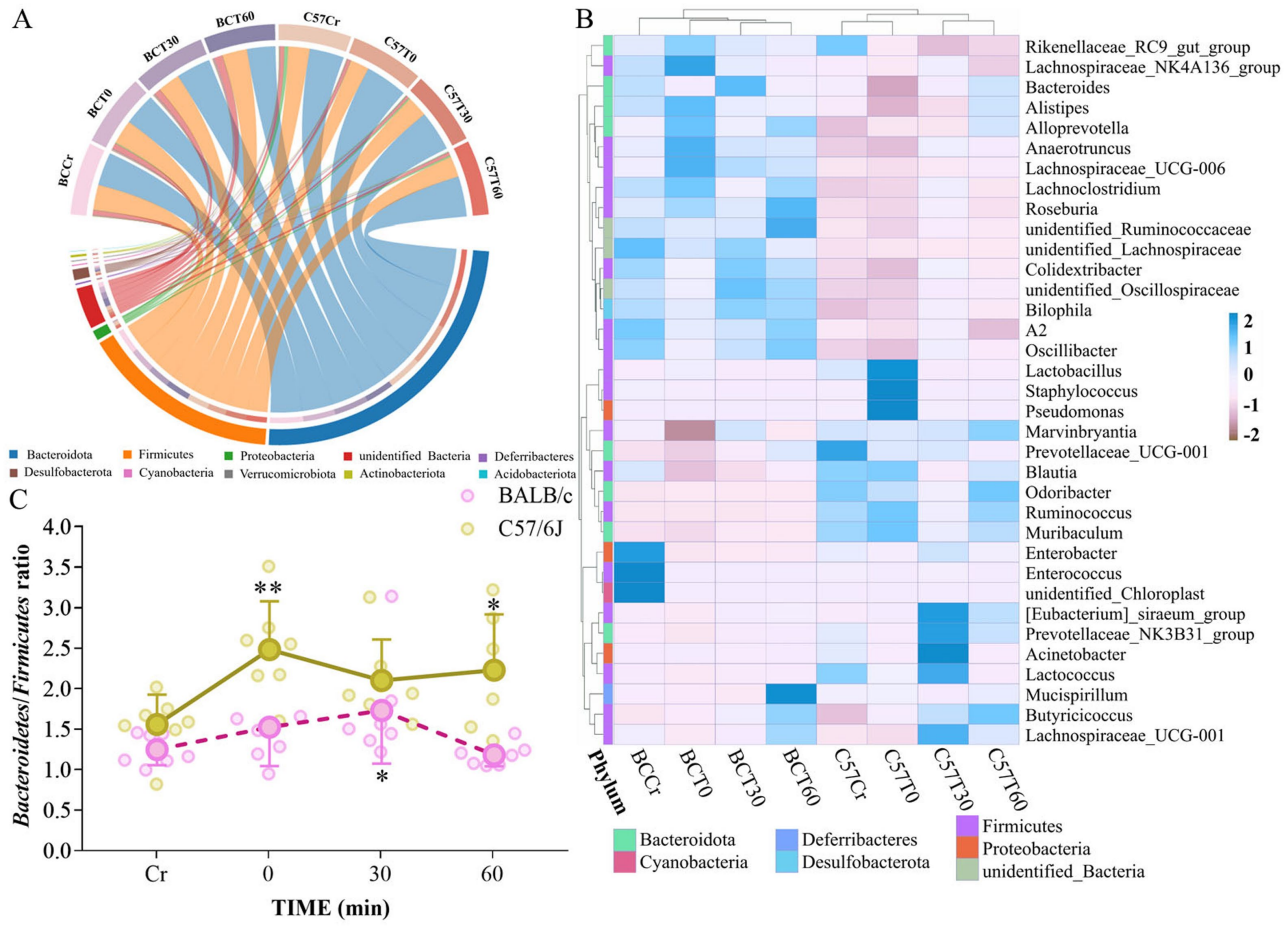
Impact of acute high-intensity exercise on compositional dynamics of gut microbiota. **(A)** Circos plot representing the top 10 most abundant phylums across groups. **(B)** Heatmap illustrating the top 35 most abundant genera across samples. **(C)** Temporal dynamics of the *Bacteroidetes/Firmicutes* ratio following acute high-intensity exercise. No significant difference was observed between the two strains at baseline (BCCr vs. C57Cr). Comparisons shown: within-strain (BCCr vs. BCT30/BCT60; C57Cr vs. C57T30/C57T60), *p < 0.05, **p < 0.01, determined by one-way ANOVA followed by LSD *post-hoc* tests.

Analysis of fecal samples by 16S rRNA sequencing revealed significant phylum-level shifts in gut microbiota composition following acute exercise in both BALB/c and C57BL/6 mice, with strain-specific variations in abundance. *Bacteroidetes, Fimicutes, Proteobacteria*, and *Deferribacteres* were the dominant bacterial phyla (Figure 2A). The identified predominant phyla, *Bacteroidetes*, *Firmicutes*, and *Proteobacteria,* are well-established major constituents of murine gut microbiota.

The top 35 most abundant genera were selected based on genus-level species annotation and abundance data from all samples. These genera were clustered at the genus and sample levels and displayed in a heatmap according to their relative abundance (Figure 2B). While certain genera showed similar abundance patterns, most exhibited pronounced inter-strain differences. Notably, temporal changes in genus abundance following exercise were strain-dependent, with contrasting trajectories observed for specific taxa (e.g., *Lactobacillus*, *Pseudomonas*, and *Staphylococcus* were enriched in C57BL/6 mice at 60 min post-exercise [C57T60], whereas *Enterococcus* and *Enterorhabdus* predominated in BALB/c mice at baseline [BCT0]).

The ratio of *Bacteroidetes* to *Firmicutes*, a critical indicator of intestinal microecology, increased post-exercise in both strains, with a more marked elevation in C57BL/6 mice (Figure 2C). This increase suggests improved intestinal health following exercise, as previous studies have associated dysregulation of the *Bacteroidetes*/*Firmicutes* ratio with metabolic disorders (e.g., obesity, diabetes).

### 3.2 Significant changes in gut microbiota alpha diversity following acute exercise

**Figure 3:**
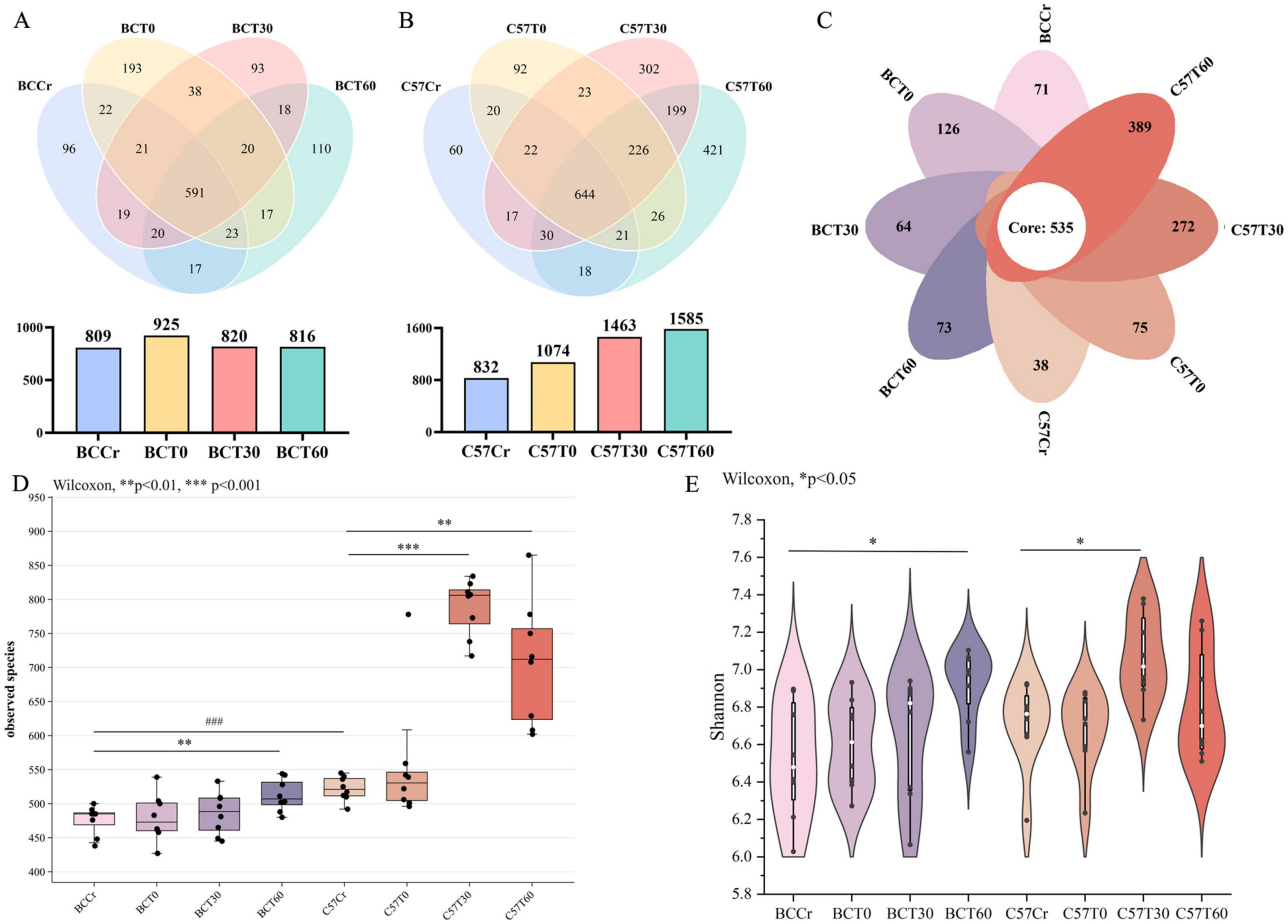
Acute high-intensity exercise significantly alters gut microbiota α-diversity. **(A,B)** Venn diagrams illustrating the average number of operational taxonomic units (OTUs) and overlapping OTUs among groups in **(A)** BALB/c and **(B)** C57BL/6 mice. **(C)** Petal diagram showing shared and unique bacterial genera across sample groups. **(D,E)** Alpha diversity as indicated by **(D)** observed species and **(E)** Shannon index across groups. Comparisons shown: within-strain (BCCr vs. BCT30/BCT60; C57Cr vs. C57T30/C57T60), ^**^p < 0.01, ***p < 0.001; and between-strain baseline (BCCr vs. C57Cr). ^###^*p* < 0.001, determined by Wilcoxon test. *p*-values were adjusted for multiple testing with the Benjamini-Hochberg FDR correction.

The average number of OTUs and shared OTUs across different groups (BCCr, BCT0, BCT30, BCT60, C57Cr, C57T0, C57T30, and C57T60) were visualized using Venn diagrams (Figures 3A,B). A total of 591 genera were shared by all BALB/c mouse groups pre- and post-exercise, with exercise eliciting dynamic genus-level changes. The BCCr group had 96 unique genera, BCT0 had 193 unique genera, BCT30 had 93 unique genera, and BCT60 had 110 unique genera (Figure 3A). In contrast, C57BL/6 mice displayed greater microbial plasticity, with 644 shared genera across groups; however, the C57Cr group had 60 unique genera, C57T0 had 92 unique genera, C57T30 had 302 unique genera, and C57T60 had 421 unique genera (Figure 3B). Petal plot analysis further confirmed these strain-specific differences, revealing a significantly higher number of unique genera in C57T30 and C57T60 groups compared to other groups (Figure 3C). These results indicate that acute aerobic exercise has the most substantial impact on gut microbiota composition in C57BL/6 mice at 30 and 60 min post-exercise.

Alpha diversity analysis was performed to assess changes from baseline (sham-exercise) within each strain. To comprehensively characterize community structure, we used observed species (richness) and the Shannon index (richness and evenness) (Eitan et al., 2024). The observed species indicated that the BCT60 (*p* < 0.01), C57T30 (*p* < 0.001), and C57T60 (*p* < 0.01) groups had significantly greater numbers of observed species (Figure 3D), suggesting the emergence of new genera post-exercise. At baseline, C57BL/6 mice harbored significantly more OTUs than BALB/c mice (*p* < 0.001), and this strain difference persisted across all post-exercise time points, with a particularly notable expansion of novel genera in the C57T30 and C57T60 groups. Moreover, substantial differences in Shannon index were identified between groups: BCCr vs. BCT60 (*p* < 0.05) and C57Cr vs. C57T30 (*p* < 0.05). Notably, temporal diversity patterns differed between strains; while BALB/c mice displayed increasing shannon diversity from 30 to 60 min post-exercise (BCT30 vs. BCT60), C57BL/6 mice exhibited peak diversity at 30 min (C57T30) followed by a decline at 60 min (C57T60) (Figure 3E). This decline, despite sustained high richness, reflects decreased community evenness driven by the increasing dominance of exercise-responsive genera. Overall, exercise enhanced intestinal microbial diversity in both mouse strains; however, the increase was more pronounced in C57BL/6 mice than in BALB/c mice.

### 3.3 Significant changes in gut microbiota beta diversity following acute exercise

**Figure 4:**
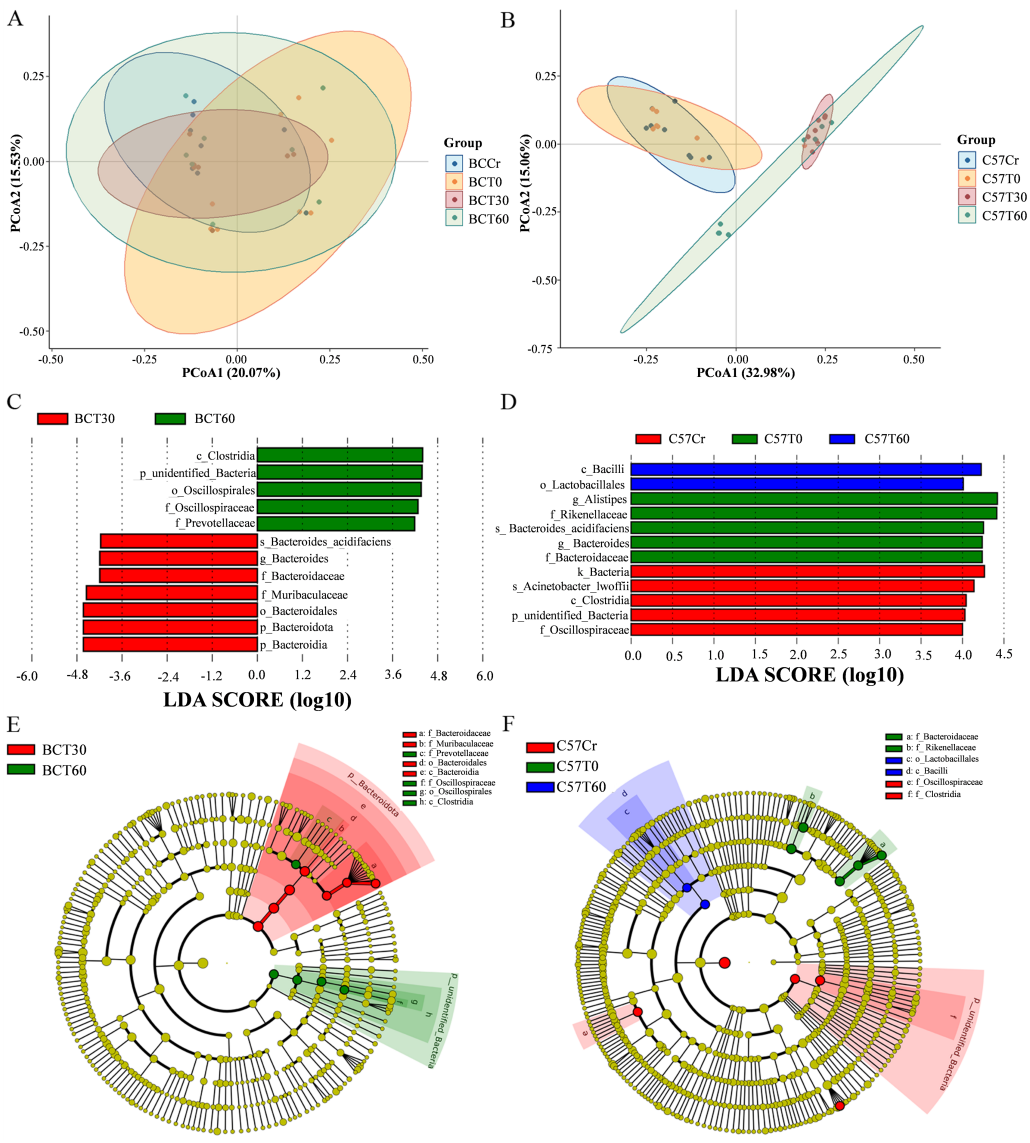
Acute high-intensity exercise significantly alters gut microbiota *β*-diversity. **(A,B)** PCoA plots based on unweighted UniFrac distances showing distinct clustering of groups in **(A)** BALB/c and **(B)** C57BL/6 mice. Ellipses represent 95% confidence intervals around group centroids. **(C,D)** LDA scores from multi-group comparison among BCCr, BCT0, BCT30, and BCT60 groups in **(C)** BALB/c mice, and among C57Cr, C57T0, C57T30, and C57T60 groups in **(D)** C57BL/6 mice. Only taxa with LDA score > 4.0 and Wilcoxon test *p* < 0.05 (adjusted by Benjamini–Hochberg FDR) are shown. **(E,F)** LEfSe identifying specific microbial signatures in **(E)** BALB/c and **(F)** C57BL/6 mice groups. The same significance thresholds as in **(C,D)** were applied.

Regarding community similarity, PCoA analysis revealed significant shifts in microbial composition in the C57T30 and C57T60 groups compared to the C57Cr group (Figure 4B). In contrast, acute exercise had a less pronounced impact on BALB/c groups (BCT0, BCT30, and BCT60) compared to the BCCr group (Figure 4A). Both ANOSIM and PERMANOVA analyses confirmed significant between-group differences in gut microbial community structure. ANOSIM analysis confirmed significant between-group differences (all R > 0), with the most substantial variations observed between mouse strains (BCCr vs. C57Cr: *p* < 0.001, R = 0.803) and across post-exercise time points (C57Cr vs. C57T0: *p < 0.05*; C57Cr vs. C57T30/T60: *p* < 0.001; BCCr vs. BCT30: *p* < 0.001; BCCr vs. BCT60: *p* < 0.05). Notably, no significant difference was observed between the BCCr and BCT0 groups (*p > 0.05*).

Additionally, LEfSe analysis revealed that at 30 min post-exercise, BALB/c mice were enriched in taxa belonging predominantly to the phylum *Firmicutes*, including *Clostridia*, *Oscillospirales*, and *Oscillospiraceae* (Figure 4C). At 60 min post-exercise, however, most enriched taxa belonged to the phylum *Bacteroidetes*, specifically *Prevotellaceae*, *Muribaculaceae*, and *Bacteroidaceae* (Figure 4C). In C57BL/6 mice, the C57T60 group showed enrichment in *Bacilli* and *Lactobacillales*, whereas *Alistipes*, *Rikenellaceae*, *Bacteroides*, and *Bacteroidaceae* were abundant in the C57T0 group (Figure 4D). Further examination via cladograms confirmed significant enrichment of *Bacteroidaceae*, *Muribaculaceae*, *Prevotellaceae*, *Bacteroidales*, and *Bacteroidia* in the BCT60 group, while *Clostridium*, *Oscillospirales*, Oscillospiraceae, and related taxa predominated in the BCT30 group (Figure 4E). In the C57T60 group, taxa such as *Bacteroidaceae*, *Rikenellaceae*, *Bacteroides*, and *Alistipes* were significantly enriched (Figure 4F). These findings suggest that acute aerobic exercise significantly promotes the proliferation of beneficial microbial taxa.

### 3.4 Comparative analysis of genus-level composition and functional shifts in BALB/c and C57BL/6 mice

**Figure 5:**
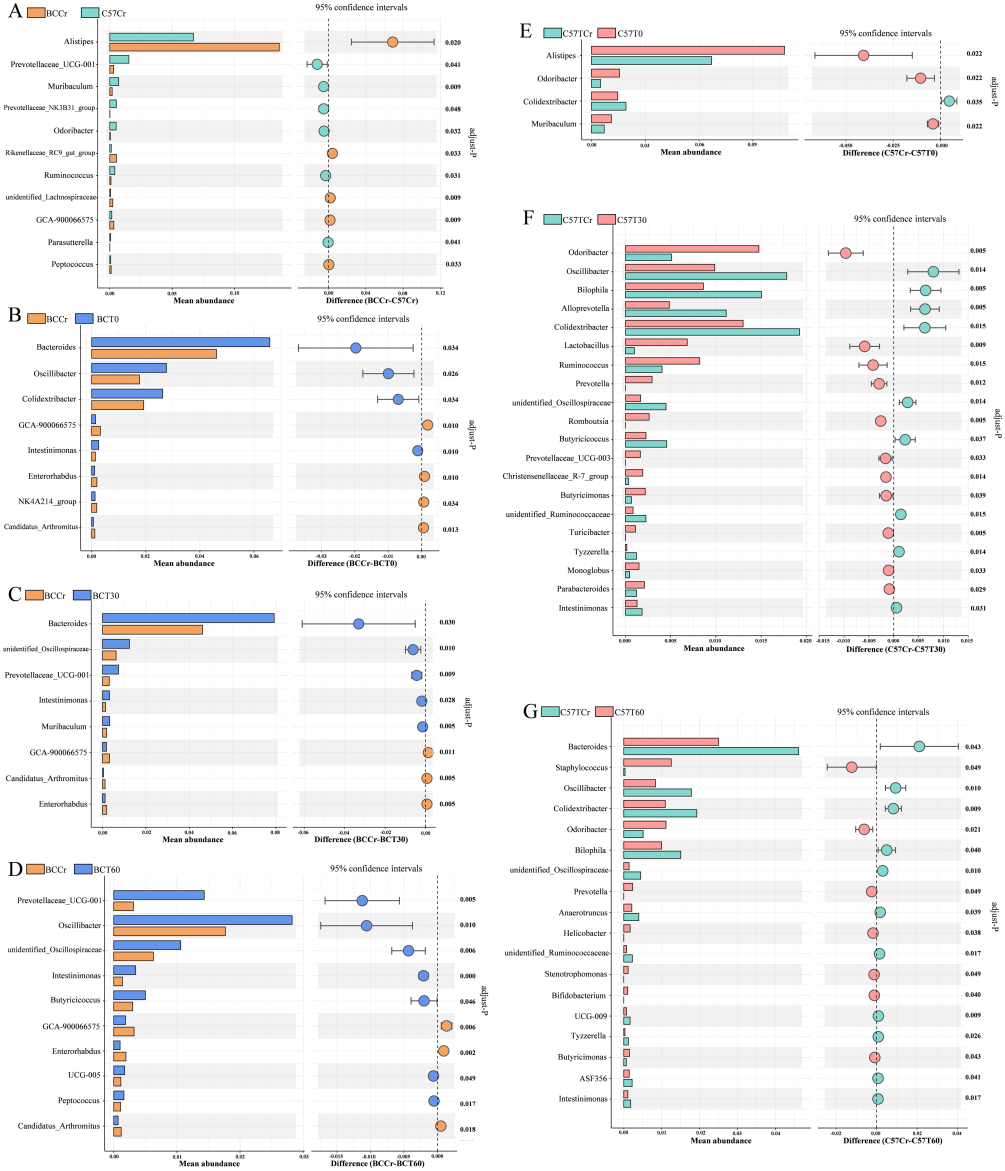
Genus-level taxonomic divergence in gut microbiota between BALB/c and C57BL/6 mice following acute high-intensity exercise. **(A–G)** Comparative genus-level profiling reveals significant differences between **(A)** BCCr vs. C57Cr; **(B)** BCCr vs. BCT0; **(C)** BCCr vs. BCT30; **(D)** BCCr vs. BCT60; **(E)** C57Cr vs. C57T0; **(F)** C57Cr vs. C57T30; and **(G)** C57Cr vs. C57T60 groups. *p*-values were adjusted for multiple testing using the Benjamini-Hochberg FDR method.

Comparative analysis between control groups (C57Cr vs. BCCr) identified 11 genera significantly differing in abundance, classified into the phyla *Bacteroidetes*, *Firmicutes*, and *Proteobacteria*. Of these, only *Alistipes* and *Prevotellaceae_UCG-001* had average relative abundances exceeding 1% across all samples (Figure 5A). Functional analysis revealed that these genera are primarily involved in energy metabolism. Specifically, *Alistipes* produces succinic acid, a substrate for the TCA cycle, and modulates immune responses through the HIF-1α/IL-1β signaling pathway.

To investigate dynamic genus-level shifts in gut microbiota following acute high-intensity exercise, eight genera with significant intergroup differences were identified immediately after exercise (BCCr vs. BCT0) in BALB/c mice at T0 (immediately post-exercise). These genera belonged to the phyla *Bacteroidetes, Actinobacteriota*, and *Firmicutes*, with only three (*Bacteroides*, *Colidextribacter*, and *Oscillibacter*) exhibiting mean relative abundances above 1% across all samples (Figure 5B). Similarly, four genera displaying significant distributional changes were identified in C57BL/6 mice (C57Cr vs. C57T0), belonging to the phyla *Firmicutes* and *Bacteroidetes*, each having a mean relative abundance exceeding 1% (Figure 5E).

At 30 min post-exercise, BALB/c mice (BCCr vs. BCT30) showed significant distributional differences in eight genera, assigned to the phyla *Bacteroidetes*, *Firmicutes*, and *Actinobacteriota*. Among these, the dominant genus shifted towards *Bacteroides*, with only *Bacteroides* and *Oscillospiraceae* maintaining mean relative abundances above 1% (Figure 5C). In C57BL/6 mice (C57Cr vs. C57T30), 20 genera exhibiting significant intergroup variation were identified, belonging to the phyla *Bacteroidetes, Firmicutes*, and *Desulfobacterota*. *Alloprevotella*, *Lactobacillus*, *Colidextribacter*, *Bilophila*, *Oscillibacter*, and *Odoribacter* had mean relative abundances greater than 1% across samples. Remarkably, novel genera such as *Romboutsia* (0.2615%), *Prevotella* (0.2944%), and *Prevotellaceae_UCG-003* (0.1643%) emerged in the C57T30 group but were absent in controls (C57Cr) (Figure 5F). This observation supports the notion that acute exercise significantly enhances microbial diversity, though the precise mechanisms underlying these increases remain unclear.

At 60 min post-exercise, BALB/c mice (BCCr vs. BCT60) displayed 10 genera with significant distributional differences, assigned to *Bacteroidetes*, *Firmicutes*, and *Actinobacteriota*. Among these, *Oscillibacter*, *Oscillospiraceae*, and *Prevotellaceae_UCG-001* had mean relative abundances of approximately 1% or more (Figure 5D). For C57BL/6 mice (C57Cr vs. C57T60), 19 genera showing significant intergroup variations were identified, belonging to *Bacteroidetes*, *Firmicutes*, *Desulfobacterota*, and *Actinobacteriota*. The genera *Bacteroides*, *Colidextribacter*, *Bilophila*, *Oscillibacter*, *Odoribacter*, and *Staphylococcus* consistently exhibited mean relative abundances exceeding 1% (Figure 5G). Compared to the C57T30 group, the C57T60 group exhibited a relatively high number of significantly altered genera but fewer novel OTUs, suggesting stabilization of gut microbial composition at this time point.

Based on a comprehensive literature review of the functional analysis of the differentially abundant genera revealed alterations in immune-regulatory pathways (e.g., *Bacteroides*-associated pathways), elevated carbohydrate metabolism (e.g., *Lactobacillus*-mediated lactic acid production), and an increase in SCFA-producing bacteria (e.g., *Alloprevotella*) in both mouse strains. Collectively, these findings indicate that despite strain-specific genus-level differences, acute exercise drives functional convergence in gut microbiota through similar metabolic pathways.

### 3.5 Temporal dynamics of exercise-induced gut microbiota alterations in BALB/c and C57BL/6 mice

**Figure 6:**
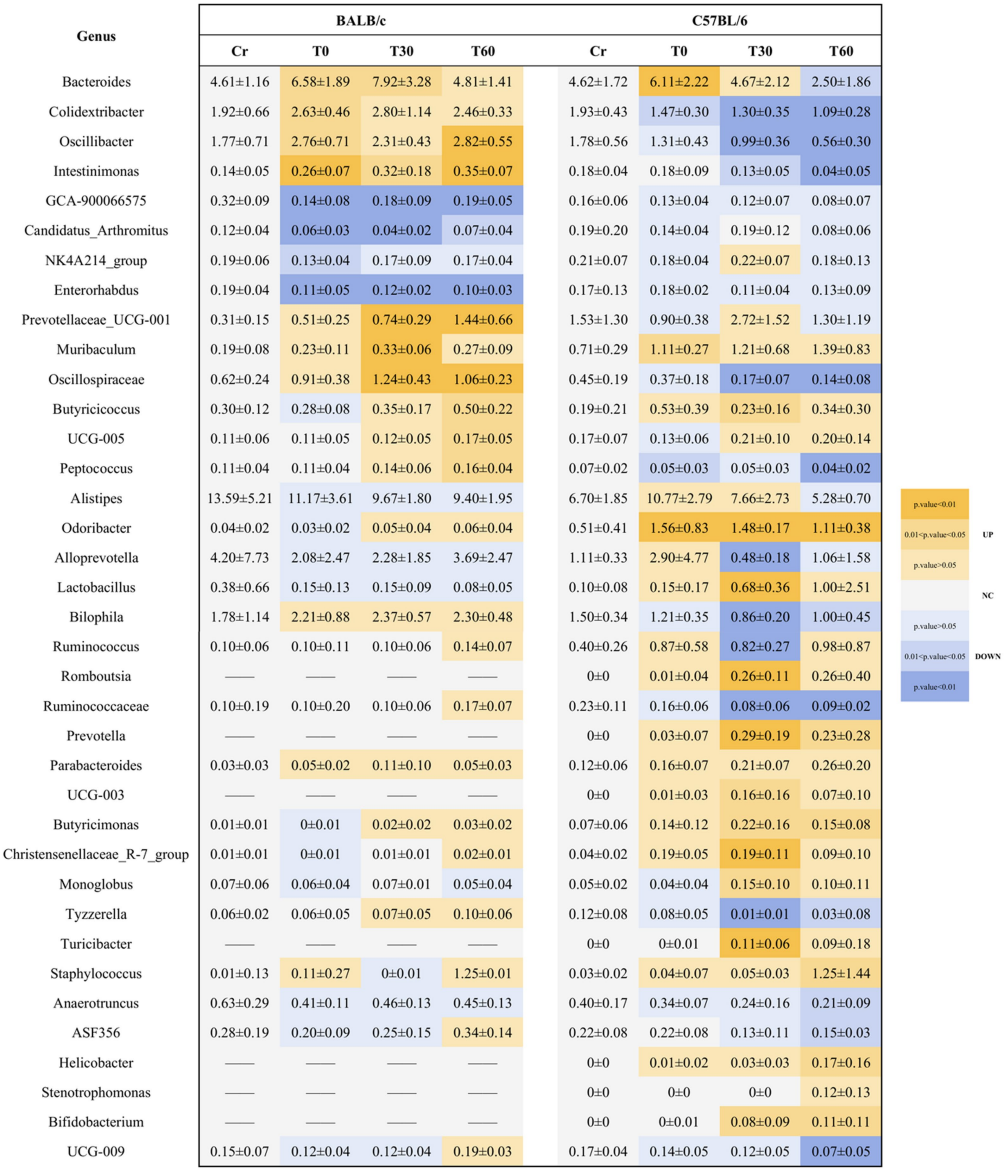
Temporal dynamics of significant genus-level changes in BALB/c and C57BL/6 gut microbiota following acute high-intensity exercise. Relative abundances for all genera shown are expressed as mean ± SEM. *p*-values were adjusted for multiple testing using the Benjamini-Hochberg FDR method. Up: Up regulated; Down: Down regulated; NC: Not changed; ——: Not delected.

We generated a chronological table summarizing genera that exhibited significant changes after exercise in both mouse strains to clarify the temporal dynamics of gut microbial responses to acute high-intensity exercise (Figure 6). Functional predictions of markedly altered genera were performed based on pathways related to metabolism, human disease, and cellular processes, and literature-derived functional annotations were incorporated to improve interpretation of genus-level shifts (Table 1).

**Table 1:** Microbiota functional profiles associated with locomotor responses in BALB/c and C57BL/6 mice.

Genus	Function summary		References
Only in BALB/c	*Peptococcus*	Pathogenic bacteria, pro-inflammatory	Sencio et al. (2022), Duan et al. (2021), and Huang et al. (2021)
	*UCG-005*	Producing butyrate, fiber-degrading, bile acids deconjugation reaction	Zhou J. et al. (2023), Li J. et al. (2022), Xu S. et al. (2022), Shi et al. (2022), Shi et al. (2023), Gao et al. (2022), Song et al. (2023), and Sun J. et al. (2022)
	*NK4A214_group*	Hydrogenation of long-chain FA	Wang et al. (2019)
	*Anaerotruncus*	Producing acetic and butyrate, fiber digestion, inflammation-associated, fermenting sugars, autoimmune and metabolic disorders	Valido et al. (2022), Wang W. et al. (2022), Gao et al. (2022), Yuan et al. (2022), Eslabão et al. (2022), García-Belenguer et al. (2021), Raimondi et al. (2021), Bailén et al. (2020), Luo et al. (2022), Bianchimano et al. (2022), Thomann et al. (2022), Yue et al. (2020), Bi et al. (2022), Song et al. (2023), Yao et al. (2020), and Yang et al. (2023a)
	*GCA-900066575*	Lipid metabolism	Zhou C. et al. (2022)
	*Candidatus_Arthromitus*	Metabolism	Yang J. et al. (2023) and Vallianou et al. (2021)
	*Enterorhabdus*	Inflammatory-related, polyphenol-degrading, lignin metabolism, bile acids metabolism	Xu W. et al. (2023), Lahtinen et al. (2023), Yin et al. (2022), Tang et al. (2021), and Lee et al. (2023)
Only in C57/6J	*Bifidobacterium*	Immunomodulatory activities	Wu Y. et al. (2023), Gavzy et al. (2023), Derrien et al. (2022), and Zafar and Saier (2021)
	*Prevotella*	Bile acid metabolism-related, detoxify superoxide radicals and tolerate reactive oxygen species, producing SCFAs	Abdelsalam et al. (2023)
	*Romboutsia*	Producing butyrate, associated with diabetes or other metabolic diseas, antioxidant capacity and glucose metabolism	Wu et al. (2021), Ye et al. (2021), Zeng et al. (2019), Du et al. (2020), and Zhang P. et al. (2023)
	*Stenotrophomonas*	Colonize immunocompromised patients	Ryan et al. (2009)
	*Helicobacter*	Peptic ulcer disease and nonulcer dyspepsia	Fischbach and Malfertheiner (2018)
	*UCG-009*	Inflammation-related	Li et al. (2019)
	*Staphylococcus*	Promote inflammation	Nat Rev Dis Primers (2018)
	*ASF356*	Producing SCFAs	Wang Q. et al. (2022)
	*Turicibacter*	Producing bacteria, producing butyrate and propionate, anti-inflammation, producing serotonin	Chi et al. (2023), Yang et al. (2022), Rahman et al. (2023), Xu Y. et al. (2022); Garrido et al. (2021), Li et al. (2017), Lynch et al. (2023), and Pirozzi et al. (2023)
	*Tyzzerella*	Producing propionate, inflammatory-related	Isipato et al. (2020), Huang et al. (2022), and Sugiyama et al. (2022)
	*Monoglobus*	producing butyrate, fiber degradation, bile acid metabolism	Lesniak et al. (2022) and Kim et al. (2019)
	*Christensenellaceae_R-7_group*	Producing butyrate, fibrolytic bacteria, closely associated with host health	Mao et al. (2023), Tian et al. (2023), Wang X. et al. (2022), Jian et al. (2022), and Bian et al. (2024)
	*Butyricimonas*	Producing butyric and isobutyric acids, bile acid metabolism-related	Otsuka et al. (2024), Bai et al. (2023), Wen et al. (2023), Liu Y. et al. (2023), Dayama et al. (2020), Wang et al. (2023), and Zhuang et al. (2020)
	*UCG-003*	Alfalfa fiber hydrolysis, producing SCFAs	Gao et al. (2022)
	*Parabacteroides*	Producing acetate, anti-inflammation, antimetabolic disease-associated, producing succinate and secondary bile acid	Ishibashi et al. (2022), Yu et al. (2022), Cui et al. (2022), Bi et al. (2022), Medawar et al. (2021), and Lei et al. (2021)
	*Prevotellaceae_UCG-001*	Fiber-degrading, producing SCFAs, activation of AMPK signaling pathway	Bian et al. (2024), Mei et al. (2023), Tao et al. (2022), Peng et al. (2022), and Li H. et al. (2022)
	*Ruminococcaceae*	Producing butyrate, cellulolytic bacterial community, modulate immunotherap	Sun et al. (2021), Grenda et al. (2022), Asare et al. (2021), Jangid et al. (2022), He J. et al. (2023), Sun Y. et al. (2022), Messaoudene et al. (2022), and Zhuang et al. (2020)
	*Ruminococcus*	Metabolic diseases, intra- and extraintestinal diseases	Crost et al. (2023)
	*Bilophila*	Producing lipopolysaccharide (LPS), promote inflammation	Huang et al. (2019), Deng X. et al. (2022), Li L. et al. (2022), He et al. (2022), Zhao et al. (2022b), Zhao et al. (2022a), Wang et al. (2021), Agudelo-Ochoa et al. (2020), David et al. (2014), and Zhuang et al. (2020)
	*Lactobacillus*	Antibacterial activities, immunomodulatory activities	Liévin-Le Moal and Servin (2014)
	*Alloprevotella*	Producing SCFAs, anti-inflammation	Derrien et al. (2022), Chen et al. (2020), Zhou X. et al. (2023), Yang et al. (2023a), and Yang et al. (2023b)
	*Odoribacter*	Producing butyrate, associated with host intestinal inflammation	Zhang Y. et al. (2023), Wang G. et al. (2022), Wang et al. (2023), Wang R. et al. (2022), Deng L. et al. (2022), and Cheng et al. (2018)
	*Alistipes*	Obesity-related, anti-inflammatory, producing butyrate, bile acid metabolism-related	Cai et al. (2023), Luo et al. (2021), Fernández et al. (2020), Huang et al. (2019), Chanda and De (2024), Liang et al. (2023), Messaoudene et al. (2022), He et al. (2020), Zhong et al. (2020), David et al. (2014), and Song et al. (2023)
Common in BALB/c and C57/6J	*Butyricicoccus*	Producing butyrate, regulating bile acids	Devriese et al. (2017), Li et al. (2023), and Zhou X. et al. (2023)
	*Oscillospiraceae*	Producing butyrate, anti-inflammation	Correa et al. (2023), Litwinowicz and Gamian (2023), Pirozzi et al. (2023), Leth et al. (2023), Van den Abbeele et al. (2022), Yang J. Y. et al. (2023), and Juárez-Castelán et al. (2022)
	*Muribaculum*	Carbohydrate metabolism, immunomodulatory screen, deconjugation and oxidation of bile acid	Wang J. L. et al. (2022), Chung et al. (2020), Medina-Larqué et al. (2022), Lee et al. (2023), Shi et al. (2023), Marion et al. (2020), Abbondio et al. (2023), Yang B. et al. (2023), Xu H. et al. (2023), and Wang T. et al. (2022)
	*Intestinimonas*	Producing butyrate, anti-obesity and anti-inflammatory, prevent obesity and improve insulin sensitivity	Zhu et al. (2024), He K. et al. (2023), Zhou Y. et al. (2022), Companys et al. (2021), Du et al. (2020), Zhuang et al. (2020), Chen et al. (2024), Cheng et al. (2023), Liu T. et al. (2023), Song et al. (2023), Yu et al. (2022), Cai et al. (2020), Bui et al. (2015), and Bui et al. (2016)
	*Oscillibacter*	Obesity-related, producing SCFAs	Newman et al. (2023), Scorletti et al. (2020), Yao et al. (2020), and Thingholm et al. (2019)
	*Colidextribacter*	Oxidative stress, anti-inflammatory and lipid deposition, producing SCFAs	Sun J. et al. (2022), Yang et al. (2023a), Yang et al. (2023b), Wu Q. et al. (2023), Liu et al. (2022), and Zhang P. et al. (2023)
	*Bacteroides*	Polysaccharides degradation, anti-inflammation, producing SCFAs, synthesizing Vitamin K	Wexler (2007), Tan et al. (2019), and Bäckhed et al. (2005)

Functional roles are inferred from published literature (references shown) and represent predictions based on taxonomy, not direct measurements from this study.

In BALB/c mice, four genera were significantly up-regulated and four were down-regulated immediately after exercise (BCT0). Among the down-regulated taxa, *Candida*, which participates in host immune regulation, showed the greatest decline (47%). At 30 min post-exercise (BCT30), five genera increased and three decreased. *Candida* abundance continued to decline, whereas genera associated with energy metabolism—particularly *Prevotellaceae_UCG*-*001*, which increased by 138%—continued to expand. By 60 min post-exercise (BCT60), ten genera were significantly altered, with three down-regulated and seven up-regulated. Most genera linked to energy metabolism and immune regulation increased further at T60 compared to T30, with the exception of *Candida*, which continued to decrease.

In C57BL/6 mice, only four genera changed significantly immediately after exercise (C57T0), with the most strongly up-regulated genus showing a 61% increase. At 30 min (C57T30), a pronounced response was observed, with 20 genera significantly altered. Most were functionally associated with energy metabolism and anti-inflammatory defense, supporting intestinal barrier protection. Among the nine down-regulated genera, *Clostridium*, *Cholera* spp., and *Tyzzerella* butyricum, taxa linked to intestinal inflammation and cardiovascular disease, showed substantial reductions (−43, −50%, and −88%, respectively). These findings suggest that C57BL/6 mice exhibit rapid reductions of inflammation-associated taxa to counteract exercise-induced stress and restore energy balance. Sixty minutes post-exercise (C57T60), 18 genera showed significant abundance changes, of which seven were up-regulated and eleven were down-regulated. However, the number of significantly altered genera at T60 was slightly lower than at T30. Compared with the T0 time point and all post-exercise phases in BALB/c mice, the C57T60 phase exhibited a notably higher number of significantly altered genera. Although still significantly different from baseline at T60, genera such as *Clostridium butyricum*, *Tyzzerella*, and *Cholera* spp. showed peak reductions at T30, whereas most genera associated with energy metabolism declined from T30 to T60. Interestingly, genera with increased abundance included both pathogenic taxa (e.g., *Staphylococcus*, *Helicobacter*, and *Stenotrophomonas*) and beneficial intestinal barrier-associated taxa (e.g., *Anaerotruncus* and *Bifidobacterium*). Despite having fewer significantly altered genera than the C57T30 group, the C57T60 group showed the broadest phylum-level diversity and the highest number of non-significantly altered genera. These results suggest that despite the presence of potentially harmful bacteria, acute exercise interventions in C57BL/6 mice promote rapid energy recovery and beneficially reshape overall gut microbial diversity and composition.

A comparative analysis between the two mouse strains revealed that certain genera present at low baseline abundances (<0.2%) in C57BL/6 mice were absent or rare in BALB/c mice. Additionally, these low-abundance genera displayed more substantial abundance fluctuations following exercise in C57BL/6 mice. In both strains, genera with similar baseline abundances often exhibited divergent post-exercise trajectories. For instance, bile-associated genera (*Bilophila*, *Intestinimonas*, and *Colidextribacter*) showed opposing abundance changes between the strains. Overall, acute exercise intervention induced a greater number of altered genera in C57BL/6 mice compared to BALB/c mice. C57BL/6 mice displayed more uniform changes across both high- and low-abundance genera, whereas significantly altered genera in BALB/c mice exhibited a relatively clustered pattern. Thus, C57BL/6 mice possess a greater capacity to mobilize gut microbiota in response to acute high-intensity exercise compared to BALB/c mice.

### 3.6 *Muribaculum* as a potential important genus responsive to exercise

The majority of genera, including *Colidextribacter*, *Oscillibacter*, Intestinimonas, and *Oscillospiraceae*, displayed opposing abundance trends following exercise, while only *Muribaculum* and *Butyricoccus* exhibited consistent changes across strains. Among all significantly altered genera identified in both mouse strains, nearly all showed divergent abundance trajectories between strains. From a functional perspective, the two mouse strains showed opposite trends for genera associated with energy metabolism; however, beneficial bacterial genera generally increased in both strains. For example, the abundance of *Bacteroides* increased significantly (*p < 0.05*) immediately following exercise in both strains and continued to rise 30 min post-exercise. Subsequently, bacterial abundance gradually decreased after 30 min, showing strain-specific patterns at 60 min. In BALB/c mice, *Bacteroides* abundance returned to baseline levels, whereas in C57BL/6 mice, abundance decreased to half of baseline levels.

**Figure 7:**
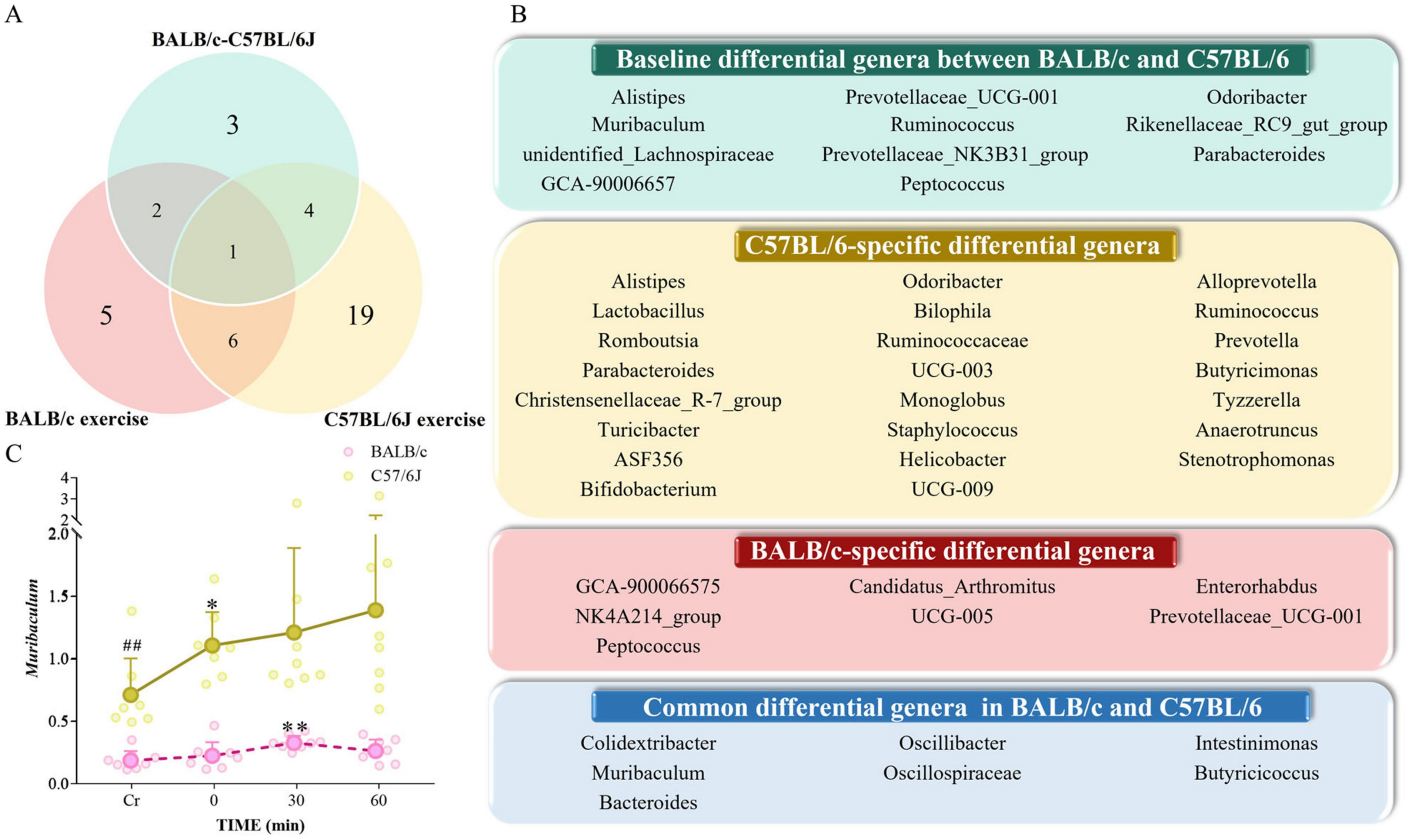
Key genus responses to acute high-intensity exercise. **(A)** Venn diagram illustrating consistent and variable genus-level responses to exercise. **(B)** Conserved and divergent genus-level exercise responses in BALB/c and C57BL/6 mice. **(C)** Temporal changes in *Muribaculum* abundance following acute high-intensity exercise. Comparisons shown: within-strain (BCCr vs. BCT30/BCT60; C57Cr vs. C57T30/C57T60), *p < 0.05, **p < 0*.01*; and between-strain baseline (BCCr vs. C57Cr). ##*p* < 0.01, determined by one-way ANOVA followed by LSD post-hoc tests.

Venn diagrams were utilized to illustrate the mean and median numbers of significantly altered genera between mouse strains following exercise (Figure 7A). The majority of significantly altered genera unique to C57BL/6 mice were associated with resistance to intestinal inflammation and energy metabolism. Probiotic bacterial abundance peaked at 30 min post-exercise and declined by 60 min post-exercise. Although changes in probiotic and pathogenic bacterial abundance were generally inversely correlated, several pathogenic taxa exhibited distinct temporal dynamics (Figure 7B). The abundance of *Candidatus_Arthromitus*, a genus involved in regulating host immune responses, decreased from immediately after exercise to 30 min post-exercise, followed by a slight increase at 60 min post-exercise. Few genera were uniquely altered in BALB/c mice; among these, *Prevotellaceae_UCG-001* and *Enterorhabdus* were associated with energy metabolism. Notably, following exercise, *Prevotellaceae_UCG-001* abundance increased more markedly than *Enterorhabdus*, which continued to decline. The pathogenic genus *Peptococcus* increased substantially 60 min post-exercise, alongside a significant rise in *Staphylococcus* abundance at the same time point (Figure 7B).

Interestingly, *Muribaculum* abundance differed significantly between mouse strains at baseline and underwent significant changes in both strains following exercise. Based on these findings, we propose *Muribaculum* as a potential important genus potentially associated with inter-strain differences in murine locomotor activity. C57Cr mice had approximately three-fold higher baseline abundance of *Muribaculum* compared to BCCr mice (Figure 7C). Post-exercise trajectories also differed between strains: *Muribaculum* abundance in BALB/c mice increased, peaking at T30 and subsequently declining at T60, whereas in C57BL/6 mice, *Muribaculum* abundance continued to rise throughout the post-exercise period (Figure 7C). Previous studies have suggested that *Muribaculum* performs diverse metabolic functions that promote nutrient breakdown and absorption. Further research is warranted, as direct associations between *Muribaculum* and exercise capacity remain unexplored.

In addition to *Muribaculum*, five other genera exhibited significant post-exercise changes in both mouse strains. We propose these genera as secondary potential taxa potentially linked to differences in locomotor performance between mouse strains (Figure 7B), despite their lack of significant baseline differences between strains. Notably, these genera displayed nearly opposite abundance trajectories between strains, likely due to genetic background differences.

## 4 Discussion

The gut microbiota, often termed the “second genome,” critically influences host metabolism and health (Min et al., 2024). Under physiological conditions, gut microbes facilitate nutrient absorption and produce essential metabolites such as folate, vitamins, and SCFAs (Clark and Mach, 2016; Huang et al., 2024). Conversely, dysbiosis contributes to chronic diseases, including obesity and hyperlipidemia (Duan et al., 2021; Quiroga et al., 2020; Illiano et al., 2020; Cuevas-Sierra et al., 2019). Exercise is increasingly recognized as a non-pharmacological modulator of gut microbial composition and metabolic function, influencing microbiota-derived metabolites and thereby promoting metabolic health and disease prevention (Barton et al., 2018; Allen et al., 2018a; Campbell and Wisniewski 2nd, 2017; Campbell et al., 2016; Bressa et al., 2017). Although the general impact of exercise on the gut microbiome has become clearer, the temporal dynamics of microbial community responses following exercise remain poorly defined. It is plausible that long-term exercise-induced alterations in gut microbiota composition arise from the cumulative effect of repeated acute perturbations—each transiently reshaping the intestinal environment and microbial activity. In this study, we conducted longitudinal analyses of gut microbiota in two widely used laboratory mouse strains to provide comprehensive insights into microbial responses to acute exercise. Since acute physiological changes in gut permeability and ion transport can occur within this timeframe, the rapid microbial shifts seen within an hour after exercise most likely reflect a combination of physical displacement due to increased gut motility and rapid functional/metabolic changes in resident microbes (Vicario et al., 2012; Leigh et al., 2023; Grosicki et al., 2023).

Overall, BALB/c mice harbored lower microbial richness than C57BL/6 mice. In BALB/c mice, acute exercise primarily induced an early enrichment of energy-associated genera, such as *Bacteroides* (Wexler, 2007; Zafar and Saier, 2021), *Colidextribacter* (Liu et al., 2022; Zhang P. et al., 2023), and *Oscillibacter* (Newman et al., 2023; Yao et al., 2020), whereas genera associated with immunomodulation, such as *Candidatus_Arthromitus* (Yang J. et al., 2023; Vallianou et al., 2021), showed marked reductions (Supplementary Table 1). These findings suggest that the immediate post-exercise microbiota shift in BALB/c mice primarily favors enhanced energy metabolism, while immunomodulatory functions may be transiently suppressed. The expansion of *Bacteroides*, *Prevotellaceae_UCG*-*001*, and *Oscillospiraceae*, particularly *Prevotellaceae_UCG*-*001* (Supplementary Table 1), which is closely linked to dietary fiber degradation and SCFA production (Bi et al., 2022; Bian et al., 2024; Mei et al., 2023; Tao et al., 2022; Peng et al., 2022; Li H. et al., 2022), likely reflects increased post-exercise energy demands. In contrast, the delayed recovery of *Candidatus_Arthromitus* suggests that immune regulatory restoration lags behind metabolic recovery. Collectively, these patterns indicate that the gut microbiota of BALB/c mice may prioritize rapid energy replenishment post-exercise, while immunoregulatory processes recover more slowly. This delayed recovery of immune-associated genera could reflect lower intrinsic energy-metabolic capacity in BALB/c mice, requiring longer periods to restore systemic energy balance after acute high-intensity exercise. Consequently, BALB/c mice exhibited lower microbial diversity (Figures 3, 4), fewer genera undergoing significant post-exercise changes, more uneven genus-level shifts (Figure 5), fewer taxa available for functional compensation, and reduced overall microbial stress resilience under exercise intervention. BALB/c mice demonstrated slower post-exercise energy recovery and a greater reliance on a limited set of energy- and inflammation-related genera, characterized by decreased beneficial genera associated with energy metabolism and immunity, alongside increased abundance of pro-inflammatory and disease-associated genera.

In contrast, gut microbiota alterations were more pronounced in C57BL/6 mice, with the number of significantly altered genera increasing over time and peaking at 60 min post-exercise. C57BL/6 mice exhibited increases in genera linked to intestinal health and energy metabolism, including *Lactobacillus* (Liévin-Le Moal and Servin, 2014), *Ruminococcus* (Crost et al., 2023), and *Prevotella* (Abdelsalam et al., 2023). Conversely, genera associated with inflammation and disease, such as *Bilophila* (Huang et al., 2019; Deng X. et al., 2022; Wang et al., 2021; Agudelo-Ochoa et al., 2020; Zhuang et al., 2020) and *Tyzzerella* (Huang et al., 2022), significantly decreased, suggesting protective microbial responses against exercise-induced inflammation. However, the abundances of both beneficial genera, such as *Bifidobacterium* (Wu Y. et al., 2023; Gavzy et al., 2023; Derrien et al., 2022; Laursen et al., 2021), and pathogenic taxa, including *Helicobacter* (Crowe, 2019) and *Staphylococcus* (Nat Rev Dis Primers, 2018), increased substantially (Supplementary Table 2). This indicates that while overall gut microbial diversity increased at 60 min post-exercise, there was also a rise in certain pathogenic bacteria, possibly reflecting changes in the gut environment following acute high-intensity exercise, although the precise mechanism remains to be determined. Collectively, these patterns suggest more efficient energy recovery in C57BL/6 mice, accompanied by reductions, rather than increases, in genera linked to cardiovascular disease and intestinal inflammation during the immediate post-exercise period, consistent with intestinal barrier protection. These findings indicate that the intestinal microbiota of C57BL/6 mice is associated with enhances intestinal health by expanding beneficial taxa and rapid post-exercise energy restoration. Following energy recovery, the simultaneous rise in both beneficial and potentially pathogenic genera reflects a complex and dynamic restructuring of the microbial community. The high number of significantly altered genera, the relatively uniform genus-level shifts, the greater availability of taxa for functional compensation, and the increased resilience of the microbial community collectively reveal a highly diverse gut flora in C57BL/6 mice under exercise intervention.

Comparative analysis of BALB/c and C57BL/6 mice following acute high-intensity exercise demonstrated marked differences in microbial response patterns. First, gut microbiota diversity was significantly higher in C57BL/6 mice than in BALB/c mice after exercise. At 30 and 60 min post-exercise, C57BL/6 mice harbored far more endemic genera (Figures 7A,B), many of which were associated with energy metabolism and resistance to intestinal inflammation. This indicates that microbial functional regulation in C57BL/6 mice is more diverse and complex during post-exercise recovery. In contrast, BALB/c mice showed increases in energy-associated genera and decreases in immunomodulatory genera, representing the dominant features of their post-exercise microbial alterations. Meanwhile, C57BL/6 mice exhibited pronounced increases in genera linked to intestinal health and energy metabolism, especially *Lactobacillus* and *Prevotella* (Supplementary Table 2), supporting the hypothesis that their gut microbiota is linked to intestinal health and accelerates energy recovery following acute exercise. Furthermore, the two strains displayed opposing abundance trends in several genera. For instance, *Colidextribacter*, *Oscillibacter*, and *Intestinimonas* were more abundant in BALB/c mice yet decreased in C57BL/6 mice (Supplementary Tables 1, 2). These contrasting dynamics suggest fundamental differences in microbial regulatory mechanisms between strains during post-exercise adaptation, likely attributable to their distinct genetic backgrounds.

Aside from these differences, the similarities in gut microbiota alterations between BALB/c and C57BL/6 mice following acute high-intensity exercise indicate a generalized effect of exercise on gut flora, theoretically supporting the health-promoting benefits of exercise through modulation of the intestinal microbiome. Functional prediction analyses further demonstrated that significantly altered *Bacteroidetes* in both mouse strains predominantly engaged in energy metabolism. This suggests gut microbes may facilitate rapid replenishment of host energy stores post-exercise by enhancing energy metabolism and nutrient absorption. The strains’ exercise responses are probably influenced by the significant baseline difference in microbiota richness (Figure 3D). Especially, lower diversity may limit the spectrum of potential changes, while higher baseline diversity may offer a greater functional repertoire for community rearrangement (Wallace et al., 2025). Therefore, it is best to think of the observed post-exercise alterations as modulations superimposed on different baseline states, with the initial community structure having some influence on the response amplitude.

We propose that *Muribaculum*, identified as a potential genus undergoing significant post-exercise changes in both mouse strains, may represent a critical genus potentially linked to inter-strain differences in locomotor capacity. Baseline abundances of *Muribaculum* varied significantly between strains and changed notably following exercise intervention (Figure 7A). Prior studies have indicated that *Muribaculum* participates in diverse metabolic activities, including lipid metabolism, SCFA synthesis, polysaccharide degradation, and nutrient digestion and absorption (Abbondio et al., 2023; Yang B. et al., 2023; Xu H. et al., 2023; Wang T. et al., 2022; Zhu et al., 2024; Bang et al., 2023). High-resolution sequencing has revealed that exercise and host genotype exert species-level selection on the gut microbiota, with different activity states harboring distinct *Muribaculum* species. This suggests that the abundance changes observed in our 16S-based study may reflect shifts in species composition rather than a bloom of a single taxon (Dowden et al., 2020). Additionally, early-life Western diet has been shown to persistently reduce *Muribaculum* intestinale abundance (McNamara et al., 2021), highlighting its environmental sensitivity. To date, *Muribaculum* has not been consistently reported as a conserved exercise-responsive genus across published studies. This may be explained by its high sensitivity to baseline microbiota composition, host genetic background, or technical factors such as sequencing resolution. However, concrete evidence linking this genus directly to exercise adaptations or locomotor performance is lacking. Therefore, future studies should employ integrative multi-omics approaches, such as metagenomics and metabolomics, to elucidate the precise roles and mechanisms of *Muribaculum* in exercise-induced metabolic adaptations and host exercise performance. These observations are limited to two mouse strains under a single acute exercise protocol. Moreover, given the profound influence of circadian rhythmicity on gut microbiota composition (Bishehsari et al., 2020; Bae et al., 2019; Matenchuk et al., 2020), future studies should be designed to address the effects of sampling time, and validation in additional models and cohorts will be essential to establish the generalizability and functional relevance of these findings.

## 5 Conclusion

Through longitudinal analysis of gut microbiota dynamics in BALB/c and C57BL/6 mice following acute high-intensity exercise, this study revealed significant temporal changes in microbial composition and functional capacity. Acute exercise markedly influenced the abundance of genera associated with energy metabolism and intestinal homeostasis. Although BALB/c and C57BL/6 mice showed divergent temporal trajectories in the response of specific genera, the central finding is the rapid and significant microbial remodeling over the post-exercise period. These observations provide a foundation for future genetic mapping studies aimed at identifying genomic loci involved in exercise–microbiota interactions.

A limitation of this study is its focus solely on acute exercise responses, leaving unanswered how chronic exercise training cumulatively shapes gut microbial communities. Additionally, although *Muribaculum* consistently emerged as a significantly altered genus post-exercise, its functional contribution to exercise adaptation remains unclear. The functional assignments presented are based on literature-derived predictions rather than direct measurements, inferring metabolic functions from taxonomy alone has inherent limitations. Future research employing integrative multi-omics approaches, including metagenomics and metabolomics, will be essential to clarify the precise mechanistic roles of *Muribaculum* and other key taxa in host energy metabolism and exercise performance. Furthermore, future studies should include extended post-exercise sampling (e.g., 3 h, 6 h, 24 h, 48 h, and 7 days) to better differentiate between acute functional shifts and sustained compositional changes. Expanding these analyses to encompass additional mouse strains and larger study cohorts would further validate the generalizability of our findings and help establish causal relationships between microbiota dynamics and exercise capacity.

## Data Availability

The RNA-seq data generated in this study have been deposited in the Gene Expression Omnibus (GEO) database under accession number GSE324758 (https://www.ncbi.nlm.nih.gov/geo/query/acc.cgi?acc=GSE324758).

## Footnotes

1
http://ccb.jhu.edu/software/FLASH/

2
http://qiime.org/scripts/splitlibrariesfastq.html

3
https://www.arb-silva.de/

4
http://www.drive5.com/usearch/manual/uchime_algo.html

## Supplementary material

SUPPLEMENTARY FIGURE S1 **(A)** Rarefaction curves of observed species for all individual samples, showing plateauing. **(B)** Rarefaction curves grouped by experimental condition (BCCr, BCT0, BCT30, BCT60, C57Cr, C57T0, C57T30, C57T60), demonstrating adequate sequencing depth across all groups.
