# Supplementary material for: Acute high-intensity exercise alters gut microbiota composition and energy metabolism in different strains of mice
Source: Front Microbiol. 2026 Apr 22;17:1790697. doi: 10.3389/fmicb.2026.1790697 (PMC13148273; doi:10.3389/fmicb.2026.1790697)
Supplement: Supplementary file 1 [file Table_1.docx]

**Suppl. Table 1 Bacterial genera with significant changes in abundance at different times after exercise in BALB/c mice**

| **Group** | **Genus** | **Baseline Abundance (%)** | **Abundance (%)** | **Change Rate** | **P-value** |
| --- | --- | --- | --- | --- | --- |
| T0 | *Bacteroides* | 4.6126 | 6.5816 | 0.43 | 0.028 |
|  | *Colidextribacter* | 1.9237 | 2.6256 | 0.36 | 0.029 |
|  | *Oscillibacter* | 1.7673 | 2.7627 | 0.56 | 0.014 |
|  | *Intestinimonas* | 0.1384 | 0.2562 | 0.85 | 0.003 |
|  | *GCA-900066575* | 0.3217 | 0.1428 | -0.56 | <0.001 |
|  | *Candidatus_Arthromitus* | 0.1182 | 0.0629 | -0.47 | 0.006 |
|  | *NK4A214_group* | 0.1881 | 0.1261 | -0.33 | 0.031 |
|  | *Enterorhabdus* | 0.1938 | 0.1099 | -0.43 | 0.002 |
| T30 | *Bacteroides* | 4.6126 | 7.9163 | 0.72 | 0.026 |
|  | *Prevotellaceae_UCG-001* | 0.3112 | 0.7410 | 1.38 | 0.003 |
|  | *Muribaculum* | 0.1890 | 0.3301 | 0.75 | 0.001 |
|  | *Oscillospiraceae* | 0.6276 | 1.2400 | 0.98 | 0.004 |
|  | *Intestinimonas* | 0.1384 | 0.3208 | 1.32 | 0.022 |
|  | *Candidatus_Arthromitus* | 0.1182 | 0.0448 | -0.62 | <0.001 |
|  | *GCA-900066575* | 0.3217 | 0.1815 | -0.44 | 0.006 |
|  | *Enterorhabdus* | 0.1938 | 0.1204 | -0.38 | 0.001 |
| T60 | *Prevotellaceae_UCG-001* | 0.3112 | 1.4434 | 3.64 | 0.002 |
|  | *Oscillibacter* | 1.7673 | 2.8247 | 0.60 | 0.005 |
|  | *Oscillospiraceae* | 0.6276 | 1.0600 | 0.69 | 0.002 |
|  | *Butyricicoccus* | 0.3002 | 0.5006 | 0.67 | 0.044 |
|  | *Intestinimonas* | 0.1384 | 0.3468 | 1.51 | <0.001 |
|  | *Candidatus_Arthromitus* | 0.1182 | 0.0686 | -0.42 | 0.015 |
|  | *Enterorhabdus* | 0.1938 | 0.1006 | -0.48 | <0.001 |
|  | *UCG-005* | 0.1130 | 0.1727 | 0.53 | 0.049 |
|  | *GCA-900066575* | 0.3217 | 0.1864 | -0.42 | 0.003 |
|  | *Peptococcus* | 0.1094 | 0.1613 | 0.47 | 0.011 |
