# Supplementary material for: Acute high-intensity exercise alters gut microbiota composition and energy metabolism in different strains of mice
Source: Front Microbiol. 2026 Apr 22;17:1790697. doi: 10.3389/fmicb.2026.1790697 (PMC13148273; doi:10.3389/fmicb.2026.1790697)
Supplement: Supplementary file 2 [file Table_2.docx]

**Supp2. Table 1 Bacterial genera with significant changes in abundance at different times after exercise in BALB/c mice**

| **Group** | **Genus** | **Baseline Abundance (%)** | **Abundance (%)** | **Change Rate** | **P-value** |
| --- | --- | --- | --- | --- | --- |
| T0 | *Alistipes* | 6.6998 | 10.7692 | 0.61 | 0.005 |
|  | *Colidextribacter* | 1.9268 | 1.4666 | -0.24 | 0.028 |
|  | *Odoribacter* | 0.5102 | 1.5642 | 2.07 | 0.009 |
|  | *Muribaculum* | 0.7137 | 1.111 | 0.56 | 0.013 |
| T30 | *Alloprevotella* | 1.1137 | 0.4847 | -0.56 | 0.001 |
|  | *Lactobacillus* | 0.0980 | 0.6834 | 5.97 | 0.002 |
|  | *Colidextribacter* | 1.9268 | 1.3010 | -0.32 | 0.007 |
|  | *Bilophila* | 1.5044 | 0.8623 | -0.43 | 0.001 |
|  | *Oscillibacter* | 1.7840 | 0.9867 | -0.45 | 0.006 |
|  | *Odoribacter* | 0.5102 | 1.4754 | 1.89 | <0.001 |
|  | *Ruminococcus* | 0.4044 | 0.8210 | 1.03 | 0.007 |
|  | *Oscillospiraceae* | 0.4483 | 0.1679 | -0.63 | 0.004 |
|  | *Butyricicoccus* | 0.4562 | 0.2263 | -0.50 | 0.029 |
|  | *Romboutsia* | 0 | 0.2615 | 1.00 | <0.001 |
|  | *Ruminococcaceae* | 0.2263 | 0.0835 | -0.63 | 0.007 |
|  | *Intestinimonas* | 0.1837 | 0.1275 | -0.31 | 0.020 |
|  | *Prevotella* | 0 | 0.2945 | 1.00 | 0.003 |
|  | *Parabacteroides* | 0.1217 | 0.2096 | 0.72 | 0.017 |
|  | *UCG-003* | 0 | 0.1644 | 1.00 | 0.023 |
|  | *Butyricimonas* | 0.0668 | 0.2189 | 2.28 | 0.032 |
|  | *Christensenellaceae_R-7_group* | 0.0356 | 0.1921 | 4.40 | 0.005 |
|  | *Monoglobus* | 0.0457 | 0.1494 | 2.27 | 0.023 |
|  | *Tyzzerella* | 0.1213 | 0.0149 | -0.88 | 0.005 |
|  | *Turicibacter* | 0.0043 | 0.1103 | 24.65 | 0.001 |
| T60 | *Bacteroides* | 4.6100 | 2.4986 | -0.46 | 0.034 |
|  | *Colidextribacter* | 1.9268 | 1.0943 | -0.43 | 0.001 |
|  | *Bilophila* | 1.5044 | 0.9986 | -0.34 | 0.025 |
|  | *Oscillibacter* | 1.7840 | 0.8395 | -0.53 | 0.002 |
|  | *Odoribacter* | 0.5103 | 1.1141 | 1.18 | 0.008 |
|  | *Staphylococcus* | 0.0343 | 1.2535 | 35.55 | 0.048 |
|  | *Oscillospiraceae* | 0.4483 | 0.1406 | -0.69 | 0.002 |
|  | *Anaerotruncus* | 0.3964 | 0.2136 | -0.46 | 0.022 |
|  | *Ruminococcaceae* | 0.1701 | 0.0941 | -0.45 | <0.001 |
|  | *ASF356* | 0.2206 | 0.1450 | -0.34 | 0.027 |
|  | *Intestinimonas* | 0.1837 | 0.1086 | -0.41 | 0.006 |
|  | *Prevotella* | 0 | 0.2338 | 1.00 | 0.047 |
|  | *Butyricimonas* | 0.0668 | 0.1503 | 1.25 | 0.034 |
|  | *Helicobacter* | 0 | 0.1692 | 1.00 | 0.020 |
|  | *Stenotrophomonas* | 0 | 0.1160 | 1.00 | 0.044 |
|  | *Bifidobacterium* | 0 | 0.1059 | 1.00 | 0.025 |
|  | *Tyzzerella* | 0.1213 | 0.0320 | -0.74 | 0.012 |
|  | *UCG-009* | 0.1675 | 0.073 | -0.56 | 0.001 |

Note: 0 indicates that the abundance of this bacterial genus is <0.01%; - denotes a negative rate of change.
