# Supplementary figures and images for: Acute high-intensity exercise alters gut microbiota composition and energy metabolism in different strains of mice
Source: Front Microbiol. 2026 Apr 22;17:1790697. doi: 10.3389/fmicb.2026.1790697 (PMC13148273; doi:10.3389/fmicb.2026.1790697)

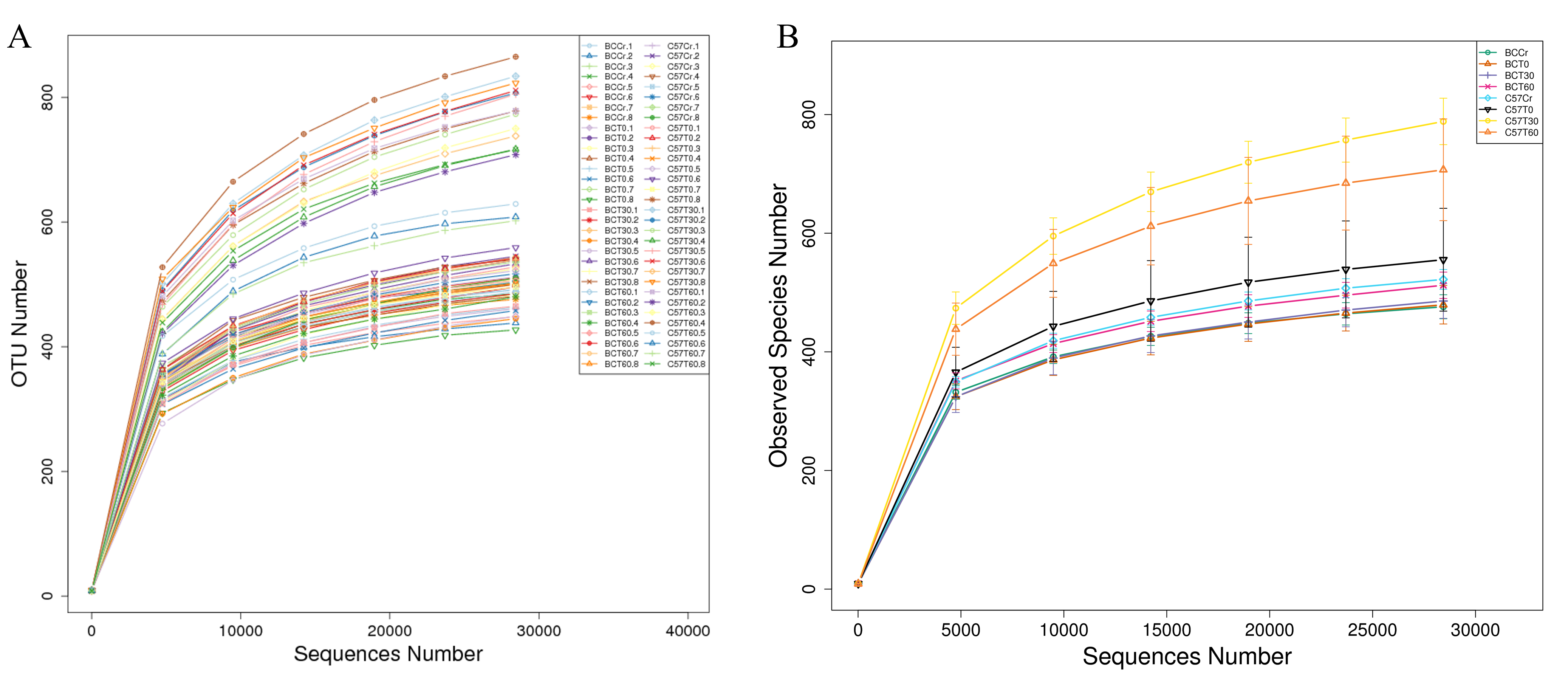

Supplement: SUPPLEMENTARY FIGURE S1 — (A) Rarefaction curves of observed species for all individual samples, showing plateauing. (B) Rarefaction curves grouped by experimental condition (BCCr, BCT0, BCT30, BCT60, C57Cr, C57T0, C57T30, C57T60), demonstrating adequate sequencing depth across all groups. [file Image_1.tif]
